# Orbital-angular-momentum fluorescence emission based on photon–electron interaction in a vortex field of an active optical fiber

**DOI:** 10.1515/nanoph-2022-0466

**Published:** 2022-12-16

**Authors:** Yan Wu, Jianxiang Wen, Fengzai Tang, Fufei Pang, Hairun Guo, Sujuan Huang, Tingyun Wang

**Affiliations:** Key Laboratory of Specialty Fiber Optics and Optical Access Networks, Joint International Research Laboratory of Specialty Fiber Optics and Advanced Communication, Shanghai Institute for Advanced Communication and Data Science, Shanghai University, Shanghai 200444, China; WMG, University of Warwick, Coventry, CV4 7AL, UK

**Keywords:** orbital angular momentum fluorescence, ring-core active fiber, vortex light source

## Abstract

We develop a model of interaction between photons and electrons in an active vortex field, which can generate a fluorescence spectrum with the characteristics of orbital angular momentum (OAM). In an active optical fiber, our findings generalize the notion of photon–electron interaction and point to a new kind of OAM-mode broad-spectrum light source, which could be interpreted in two processes: one microscopically is the excitation of OAM-carrying photons based on the photon–electron interaction; the other macroscopically is the emission and transmission of a donut-shaped fluorescence in a vortex field with a spiral phase wavefront in a ring-core active fiber. Here we present a straightforward experimental method that the emission of broad-spectrum fluorescence with an OAM feature is actualized and validated in a ring-core erbium-doped fiber. The spectrum has a broad spectral width up to 50 nm. Furthermore, four wavelengths are extracted from the fluorescence spectrum and superimposed with their corresponding Gaussian beams, from which the spiral-shaped interferograms of OAM modes in a broad spectrum are identified with high purity. The application of the OAM-based fluorescence light source may range from classical to quantum information technologies, and enable high-capacity communication, high-sensitivity sensing, high-resolution fluorescence imaging, etc.

## 1 Introduction

Structured beam has the ability to tailor light, usually exhibiting the spatial control of its amplitude, phase, and polarization [1, 2]. Therefore, it spurs a myriad of applications in optical tweezers [3], quantum optics [4, 5], optical communication [6–9], laser materials processing [10], and microscopy [11]. In recent times, structured beam has become synonymous with optical vortex beam [12]. Optical vortex beam [13–16] locally propagates along a helical trajectory and possesses a phase singularity at the beam center, resulting in a spiral phase wavefront associated with the topological charge *l*. The parameter *l* can be any integer, positive, negative and zero, which correspond clockwise and counterclockwise phase helices and Gaussian beam, respectively. It also designates the number of twists that the beam’s phase wraps around the optical axis in one period and the corresponding order of orbital angular momentum (OAM) carried. Due to the extra degree of freedom for light manipulation, vortex beam has also received tremendous attention in OAM multiplexing [17, 18], optical vortex sensing [19], and OAM-based quantum communication networks for quantum memories [20–22]. Single-wavelength laser sources were dominantly utilized for the direct generation of OAM modes [23–25], whereas the research on broadband light sources is rarely reported, and the broad-fluorescent light sources have huge advantages in several fields, such as photoluminescence imaging, i.e., they enable increased spatial resolution beyond the diffraction limit [26, 27], quantum information, i.e., the utilization of twisted photons as alphabets to encode information beyond one bit per single photon [5], and so on. Recently, Bahari et al. [28] have proposed a compact and integrated OAM light source using a structured semiconductor on a magnetic substrate based on the photonic quantum Hall effect, which can produce a fluorescence spectrum of large OAM. However, the research on OAM fluorescence emission in an active optical fiber has not been reported still. In particular, a fiber-based light source is bestowed with remarkable characteristics of easily accomplished, low loss, and fluorescence emission with a broad and flat spectrum, which can be applied to tunable OAM laser sources.

A ring-core fiber (RCF) is tailored to stably support and transmit OAM modes. In 2009, Ramachandran et al. [29] demonstrated that RCFs have a high contrast between the ring-core and cladding refractive index (RI), which increases the mode effective index separation, hence reducing induced crosstalk. Also, the RI profile closely matches the donut shape of the intensity profile of the vortex field. Thus, RCFs have been extensively used in vortex beams in place of solid-core fibers. In 2015, Zhang et al. [30] reported that the interface between the ring core and cladding needs to be smooth rather than step-type abrupt, which can eliminate the OAM mode purity impairment and intrinsic cross talk caused by spin–orbit coupling. Therefore, graded-index RCFs are commonly used to investigate vortex beam excitation and transmission. Recently, by conducting vortex modes with RCFs, OAM multiplexing could realize larger capacity and higher rate in long-haul fiber-optics communication [31–33].

In this paper, we establish a model of photon-electron interaction in an active optical fiber, and accordingly propose a method of OAM mode fluorescence emission to verify it experimentally. Based on an in-house made ring-core erbium-doped fiber (RC-EDF), which is utilized as a gain medium, a broad-spectrum fluorescence with OAM characteristics has been demonstrated in a vortex field of the RC-EDF.

## 2 Fabrication and properties of the RC-EDF

**Figure 1:**
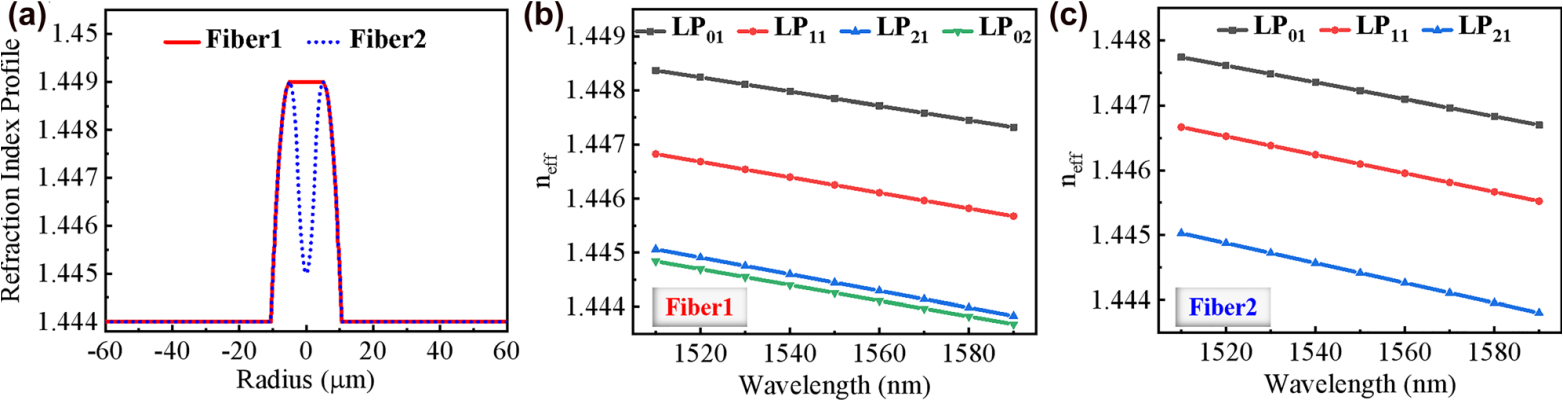
Optical fiber structural design and simulation. (a) RI profile of the designed fibers; calculated *n*_eff_ of (b) Fiber1 and (c) Fiber2.

**Figure 2:**
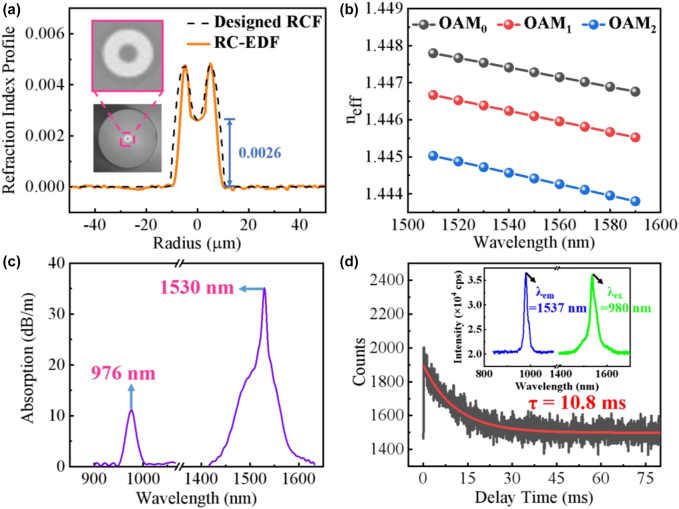
Parameters and properties of the fabricated RC-EDF. (a) RI profile (inset: cross section); (b) calculated *n*_eff_ of OAM_0_ and OAM_1_ modes versus the wavelength; (c) Absorption spectrum of OAM_1_ mode; (d) Fluorescence decay curve (inset: excitation-emission spectra).

Conventional few-mode fibers cannot suppress the radial higher-order modes, which will lead to more crosstalk between each mode. Since the ring-shaped propagation region closely matches the OAM annular intensity distribution, RCFs are considered as a special OAM-transmission fiber [34]. As shown in Figure 1(a), Fiber1 is a typical few-mode fiber which can support four modes (LP_01_, LP_11_, LP_21_, and LP_02_ modes), whose effective mode refractive index (*n*_eff_) is depicted in Figure 1(b). Under the same geometric parameters, the refraction index and the *n*_eff_ of an RCF with a *n*_dip_ of 1.445 are shown as Fiber2 in Figure 1(a) and (c), respectively. Comparing these two fibers, it can be noted for the Fiber2, there is not a radial mode (LP_02_ mode) supported, though the *n*_eff_ difference (∆*n*_eff_) between each mode is larger than that of the Fiber1. Through calculation, when the refraction index difference (∆*n*) between the dip (*n*_dip_) and cladding is less than 0.003, the radial mode can be suppressed for low-crosstalk OAM transmission. Therefore, considering the fiber fabrication condition, the fiber parameters are designed and fabricated as the black dashed curve and orange solid curve in Figure 2(a). One can see that their profiles can match well.

The active RC-EDF was fabricated by the atomic layer deposition (ALD) doping technique within the combination of the modified chemical vapor deposition (MCVD) [35, 36]. First, a thick silica soot layer was deposited by MCVD, and then Er dopants were introduced into the soot layer by ALD, where the dopant concentrations were controlled through regulating the flow rate of Er precursors. With the passing of Ge and Si chloride precursors through the tube on the MCVD lathe, the doped layer was consolidated to a co-doped active layer at high temperatures. The last deposition process was to produce a pure silica layer inside the tube in order to obtain a low RI profile in the fiber perform core. Other remaining procedures were the same as above involving fabrication of preform and optical fiber drawing.

Figure 2(a) shows the cross section and RI profile of RC-EDF. It has a *D*_core_ = 7.5 μm, a *D*_ring_ = 15.8 μm, and a *D*_cladding_ = 125.8 μm with the RI difference of 0.005 between the ring and cladding layers. Full-vectorial finite element modeling solutions of Maxwell’s equations show that high-order modes exist in the RC-EDF, and the calculated *n*_eff_ are plotted in Figure 2(b) as a function of wavelength. Since the ring-core structure and RI profiles with a dip of the fiber, the first-order OAM mode with the donut-shaped optical field distribution can stably transmit along them. Figure 2(c) shows the absorption spectrum of OAM_1_ mode at ∼976 nm and ∼1530 nm. In addition, the excitation-emission spectra and fluorescence decay curve of the RC-EDF are measured by a fluorescence spectrophotometer (FLS-980, Edinburgh, England), as shown in Figure 2(d), where the excitation and emission peaks are located at 980 nm and 1537 nm, respectively. And the fluorescent lifetime of erbium ion at 1537 nm in RC-EDF is 10.8 ms under 980 nm excitation.

## 3 Theoretical model of the excitation of OAM-carrying photons in an active fiber

**Figure3:**
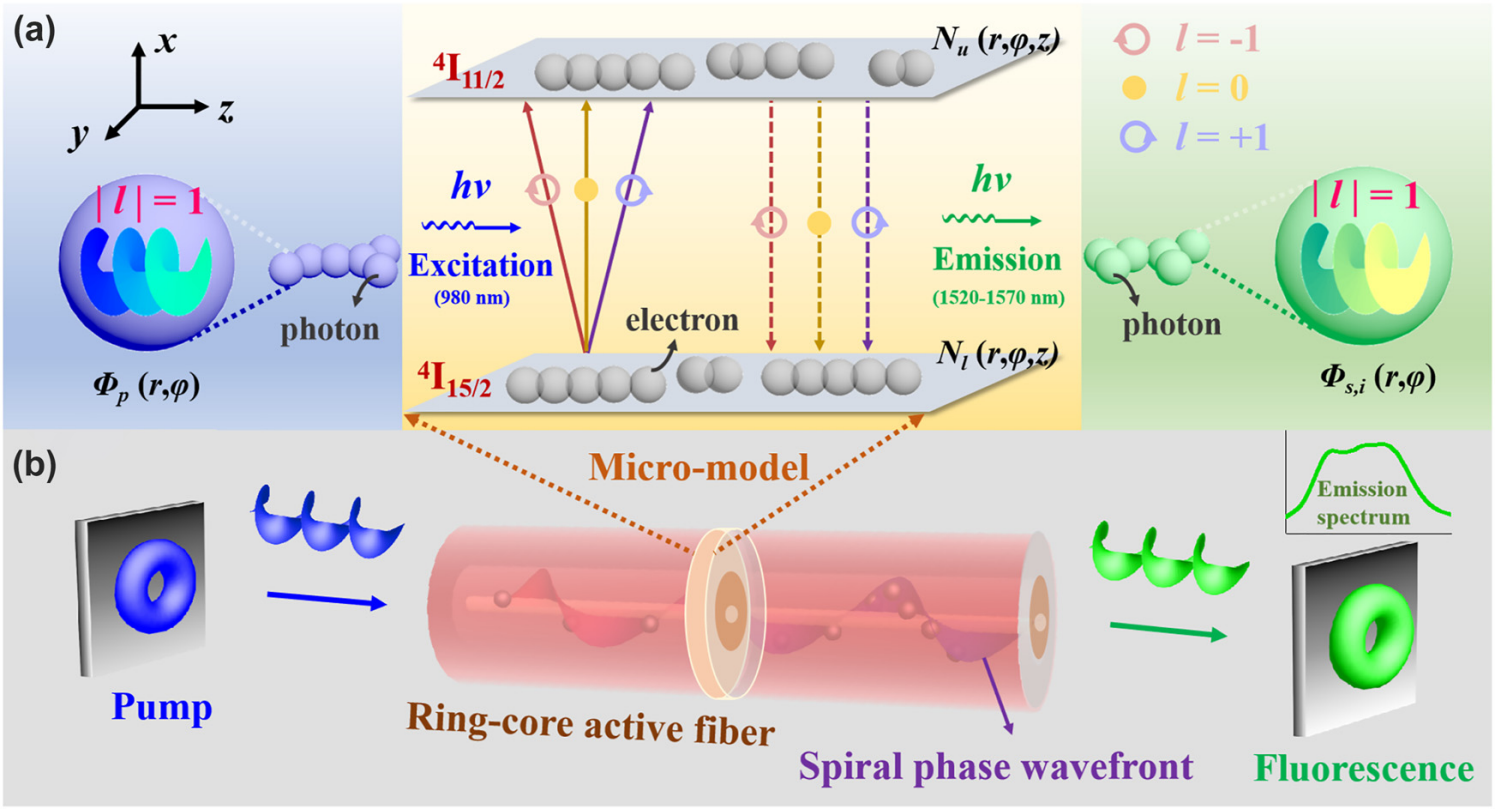
OAM fluorescence emission process in a ring-core active optical fiber. (a) An energy-level structure model of OAM-carrying photons excitation based on the photon–electron interaction: A two-level system of erbium ions consists of energy levels ^4^I_15/2_ and ^4^I_11/2_. When the OAM-carrying photon *hν* at 980 nm is utilized to pump the active fiber, the electrons in erbium ions will be excited from the ground state energy level ^4^I_15/2_ to ^4^I_11/2_. Then, the excited electrons will re-transition back to the ground state ^4^I_15/2_ in the form of spontaneous emission, during which the same amount of photons are released to form fluorescence around 1550 nm and still carry OAM in the active fiber. Especially, topological charge *l* of the initial photons carried is equal to that of the released photons (*l* = −1, 0, and +1 correspond to the line in light red, dark yellow, and purple, respectively). (b) Formation and transmission of fluorescence in a vortex field with a spiral phase wavefront in the ring-core active optical fiber: When a vortex pump beam is injected into the active fiber, a vortex field of a ring-shaped distribution will be excited with a spiral phase wavefront. Due to the ring-core structure of the active fiber, a donut-shaped fluorescence emission is implemented.

We establish an OAM-fluorescence-emission model in a ring-core active optical fiber, as shown in Figure 3. When a vortex pump beam is injected into the active fiber, the excited optical field will also have a ring-shaped distribution with a spiral phase wavefront, so called a vortex field. Under the vortex field, a donut-shaped fluorescence distribution is correspondingly achieved in the active fiber due to its ring-core structure, as shown in Figure 2(b). To gain a deeper physical insight into the established OAM-based fluorescence emission, one has to involve the particle considerations, thereby treating the formation of the vortex field in an active fiber as a photon–electron interaction process by taking an infinitesimal element on the cross section of the fiber. In this case, a two-level system of erbium ions is composed of energy levels ^4^I_15/2_ and ^4^I_11/2_, as shown in Figure 3(a). When the OAM-carrying photon *hν* in vortex pump field *Φ*_p_ (*r*,*φ*) is launched into the active fiber, the electrons in erbium ions will be excited from the ground state energy level ^4^I_15/2_ to the excited state level ^4^I_11/2_. Subsequently, the excited electrons in erbium ions with the concentration of *N*_u_ (*r*,*φ*,*z*) will further re-transition from the energy level ^4^I_11/2_ back to the ground state ^4^I_15/2_ in the form of spontaneous emission, during which the same amount of photons could be released (i.e., vortex fluorescence field *Φ*_s,*i*_ (*r*,*φ*)) and still carry OAM in the active fiber. The dynamics of the photon–electron interaction with vortex fields in the two-level system can be described with rate and power propagation equations as

(1) $$\frac{\text{d}P_{\text{p}}\left(z\right)}{\text{d}z}=-P_{\text{p}}\left(z\right)\overset{2\pi }{\underset{0}{\int }}\overset{a}{\underset{0}{\int }}\sigma_{\text{ap}}N_{\text{l}}\left(r,\varphi ,z\right)\Gamma_{\text{p}}\left(r,\varphi \right)r\text{d}r\text{d}\varphi ,$$

(2) $$\begin{array}{ll} \frac{\text{d}P_{\text{s},i}\left(z\right)}{\text{d}z} & =P_{\text{s},i}\left(z\right)\overset{2\pi }{\underset{0}{\int }}\overset{a}{\underset{0}{\int }}\left[\sigma_{\text{e}}\left(\nu_{\text{s},i}\right)N_{\text{u}}\left(r,\varphi ,z\right)-\sigma_{\text{a}}\left(\nu_{\text{s},i}\right)N_{l}\left(r,\varphi ,z\right)\right]\\ & \;\times \Gamma_{\text{s},i}\left(r,\varphi \right)r\text{d}r\text{d}\varphi +2h\nu_{\text{s},i}\left(\frac{\Delta \nu_{h}}{n}\right)\overset{2\pi }{\underset{0}{\int }}\overset{a}{\underset{0}{\int }}\sigma_{\text{e}}\left(\nu_{\text{s},i}\right)N_{\text{u}}\left(r,\varphi ,z\right)\\ & \;\times \Gamma_{\text{s},i}\left(r,\varphi \right)r\text{d}r\text{d}\varphi \end{array}$$

where *P*_p_ and *P*_s_ are powers of the pump photon and fluorescence photon, and *ν*_p_ and *ν*_s,*i*_ are the optical frequencies of pump and the *i*th fluorescence photons. The two optical frequencies are related to their respective wavelengths *λ*_p_ and *λ*_s,*i*_, and can be expressed as *ν* = *c*/*λ* where *c* is the speed of light. *h* is the Planck constant and Δ*ν*_*h*_ is the sampling interval of the fluorescence with the number of *n*. Γ_p_ and Γ_s,*i*_ are the power overlap integrals of the pump and fluorescence photons that are related to the intensity distribution of the corresponding optical field *Φ*_p_ (*r*,*φ*) and *Φ*_s,*i*_ (*r*,*φ*), respectively, which can be expressed as

(3) $$\Gamma_{\text{p}}\left(r,\varphi \right)=\frac{\Phi_{\text{p}}\left(r,\varphi \right)}{\overset{2\pi }{\underset{0}{\int }}\overset{\infty }{\underset{0}{\int }}\Phi_{\text{p}}\left(r,\varphi \right)rdrd\varphi },$$

(4) $$\Gamma_{\text{s},i}\left(r,\varphi \right)=\frac{\Phi_{\text{s},i}\left(r,\varphi \right)}{\overset{2\pi }{\underset{0}{\int }}\overset{\infty }{\underset{0}{\int }}\Phi_{\text{s},i}\left(r,\varphi \right)rdrd\varphi }.$$

*N*_l_ (*r*,*φ*,*z*) and *N*_u_ (*r*,*φ*,*z*) are the doping concentration of erbium ions at the lower and upper energy level in the RC-EDF, respectively, which can be expressed as

(5) $$N_{\text{u}}\left(r,\varphi ,z\right)=\frac{\left[\frac{1}{h\nu_{\text{p}}}P_{\text{p}}\left(z\right)\sigma_{\text{ap}}\Gamma_{\text{p}}\left(r,\varphi \right)+\frac{1}{h\nu_{\text{s},i}}P_{\text{s},i}\left(z\right)\sigma_{\text{a}}\left(\nu_{\text{s},i}\right)\Gamma_{\text{s},i}\left(r,\varphi \right)\right]N_{0}\left(r,\varphi ,z\right)}{\frac{1}{h\nu_{\text{p}}}P_{\text{p}}\left(z\right)\sigma_{\text{ap}}\Gamma_{\text{p}}\left(r,\varphi \right)+\frac{1}{h\nu_{\text{s},i}}P_{\text{s},i}\left(z\right)\left(\sigma_{\text{a}}\left(\nu_{\text{s},i}\right)+\sigma_{\text{e}}\left(\nu_{\text{s},i}\right)\right)\Gamma_{\text{s},i}\left(r,\varphi \right)+\frac{1}{\tau }},$$

(6) $$N_{0}\left(r,\varphi ,z\right)=N_{\text{u}}\left(r,\varphi ,z\right)+N_{\text{l}}\left(r,\varphi ,z\right),$$

where *N*_0_ (*r*,*φ*,*z*) and *τ* are the total doping concentration and lifetime of erbium ion in the RC-EDF. *σ*_ap_, *σ*_a,_ and *σ*_e_ are the absorption cross-sectional area of pump photons, and the absorption and emission cross-sectional area of fluorescence photons, respectively.

**Figure 4:**
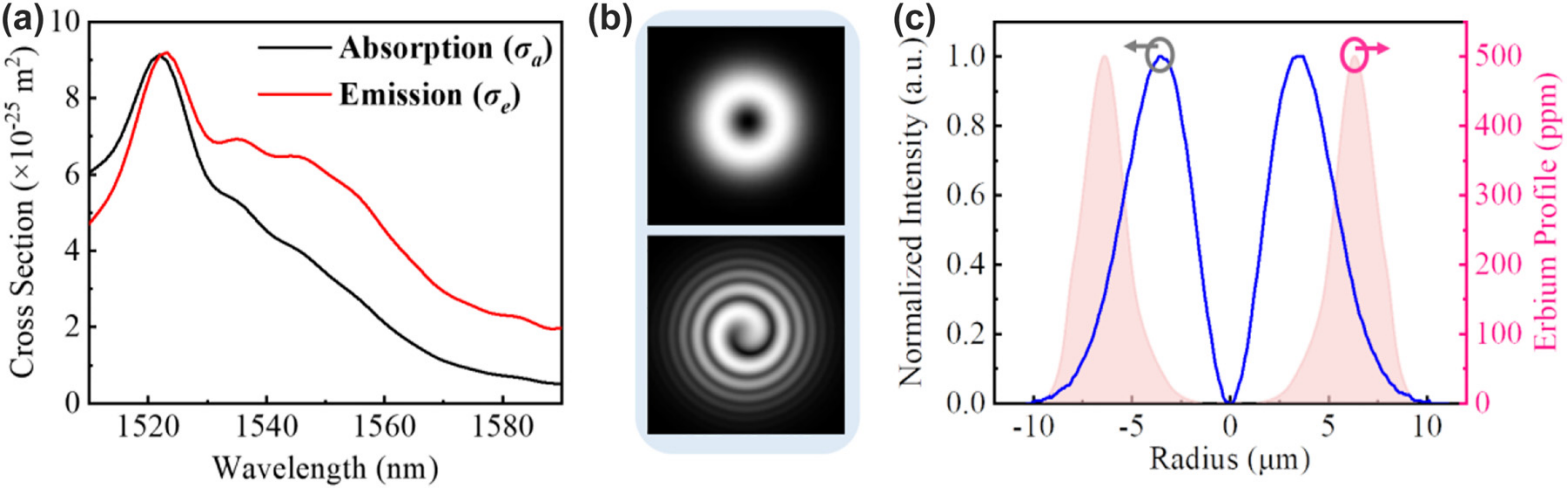
Simulated parameters of the RC-EDF. (a) Absorption and emission cross-sectional area of fluorescence photons with the OAM_1_ mode. (b) Mode pattern and interferogram, (c) normalized intensity of OAM_1_ mode at the wavelength of 980 nm, and the erbium concentration.

**Figure 5:**
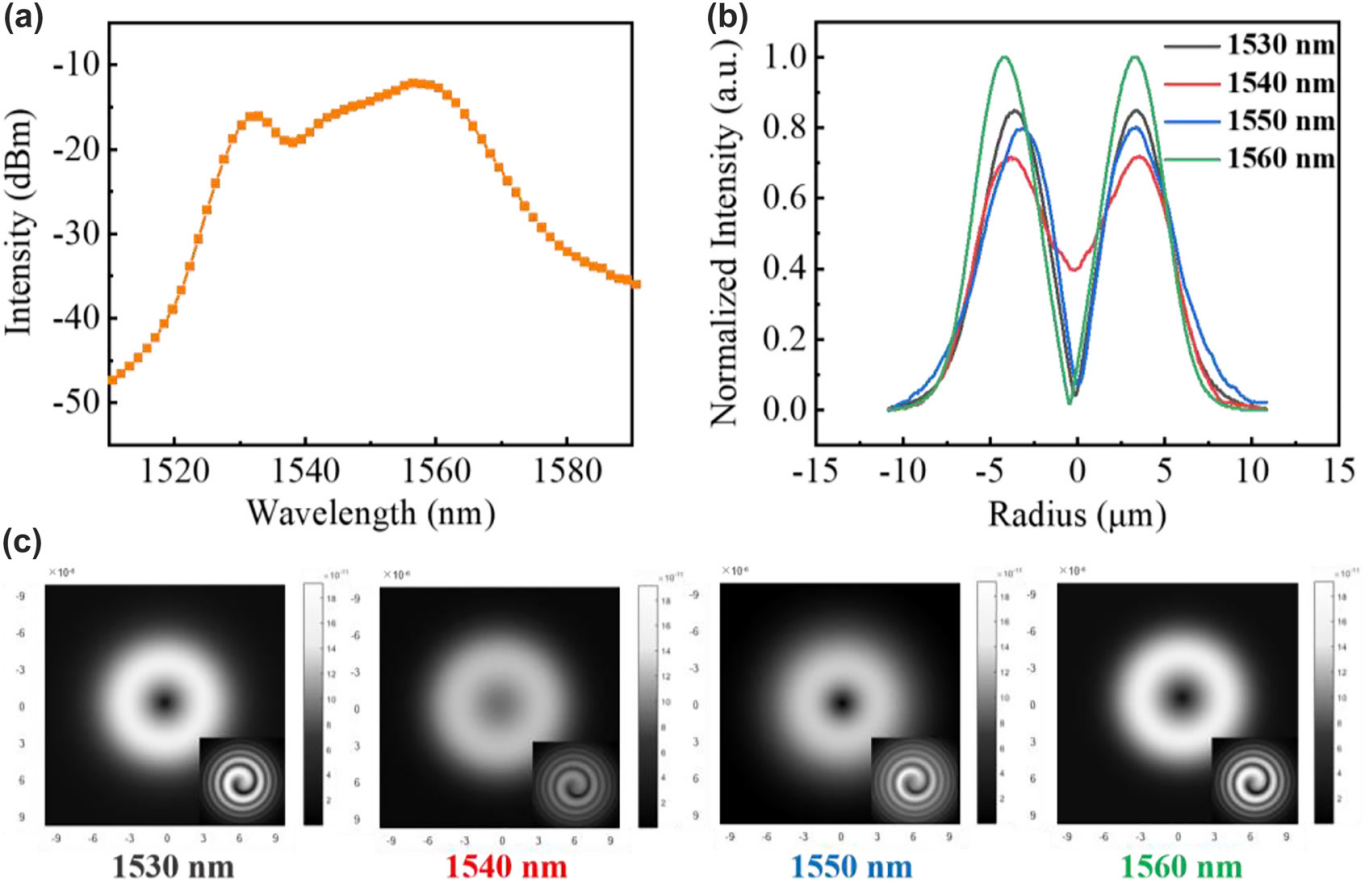
Theoretically OAM fluorescence emission in the RC-EDF. (a) Fluorescence spectrum. (b) Normalized intensity, (c) mode pattern and interferogram of OAM_1_ mode at the wavelength of 1530, 1540, 1550, and 1560 nm.

Figure 4 shows the simulated parameters of the RC-EDF. Figure 4(a) is the absorption and emission cross-sectional area of fluorescence photons with the OAM_1_ mode in the range of 1510–1590 nm. Figure 4(b) shows the vortex mode of pump photons, which presents a donut shape and the interferogram shows a spiral stripe with the topological charge of +1. The normalized profile of the vortex pump mode is described as the blue curve in Figure 4(c), and the pink region shows the erbium concentration in the RC-EDF, which also exhibits an annular distribution. The model is solved using the method of the 4th-order Runge-Kutta calculation [37]. The simulated fluorescence-emission intensity in the range of 1510–1590 nm is shown in Figure 5(a). The normalized profiles and mode patterns of the vortex fluorescence mode at 1530, 1540, 1550, and 1560 nm are depicted in Figure 5(b) and (c). Additionally, the unclear of the vortex image at 1540 nm is probably due to the strong absorption of the RC-EDF. Generally, EDF has strong absorption in the range of 1530–1540 nm, while the strongest-intensity luminescence is at near 1530 nm, which can offset some of the effects of absorption. Therefore, the fluorescence intensity near 1540 nm is weaker. At the same time, in the process of fluorescence emission, there also exist some energy-loss processes, such as Er^3+^–Er^3+^ interaction, Er^3+^-host materials interaction, phonon decay, consumption of thermal radiation, etc. [38, 39]. Those are all regarded as noise with chaotic states, which will affect the luminous efficiency. One can see that the OAM fluorescence with the topological charge of +1 is generated in accordance with the pump mode. Especially, when the pump photons carry OAM of |*l*| = 1, the transitions from level ^4^I_15/2_ to ^4^I_11/2_ are *l* = −1 and +1, as marked by the solid line in light red and purple in Figure 3(a), respectively. Then, the transitions from level ^4^I_11/2_ to ^4^I_15/2_ are correspondingly *l* = −1 and +1 by the dashed line in the same colors. The released fluorescence photons will carry OAM of *l* = −1 and +1, which correspond to the topological charge of the pump beam. In the following sections, we will further demonstrate the OAM-carrying fluorescence emission experimentally based on the formation of a vortex field in a homemade RC-EDF.

## 4 The method of OAM fluorescence emission

### 4.1 Excitation of the OAM pump with a mode selective coupler

**Figure 6:**
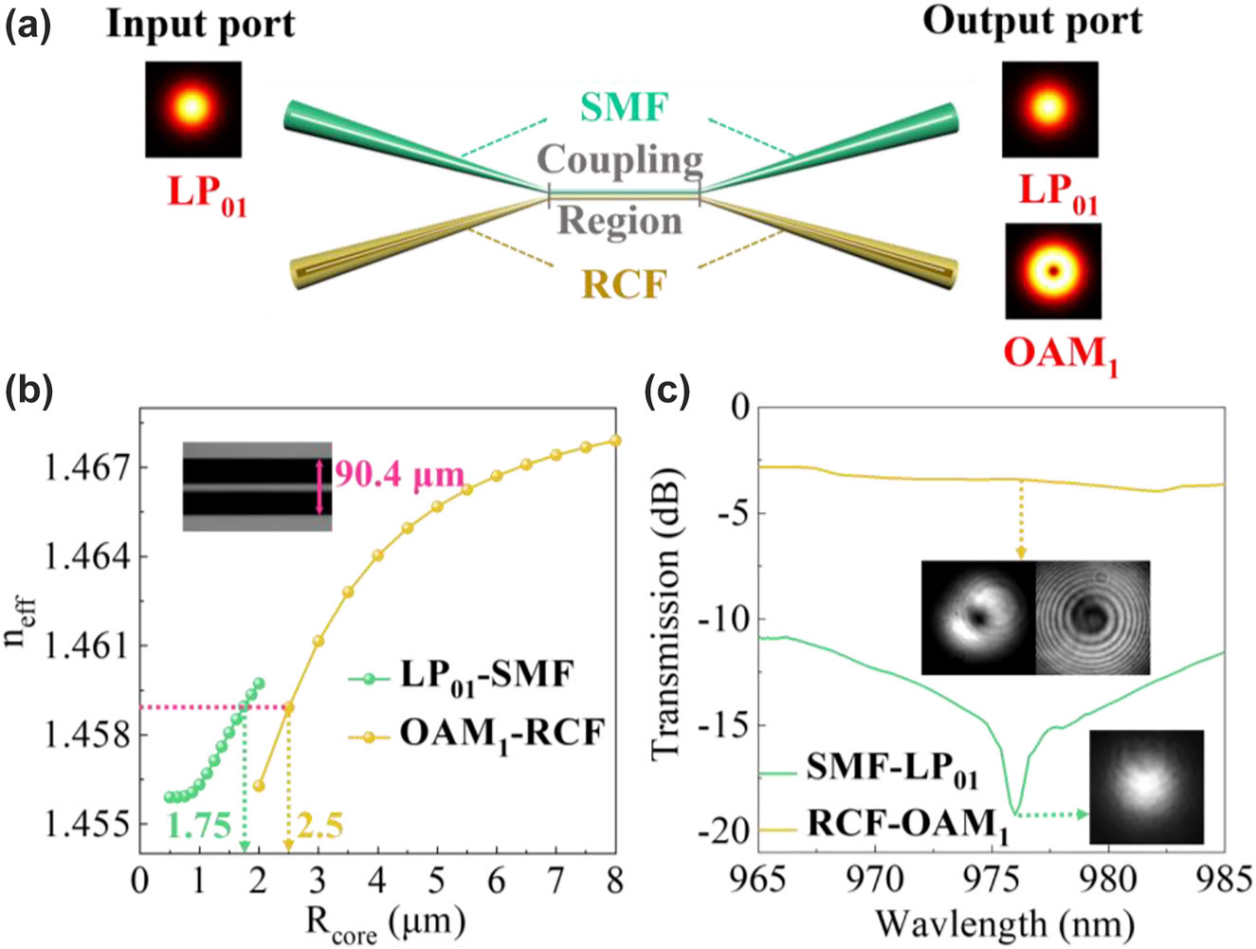
Theoretical modeling and practical performance of MSC. (a) Schematic setup of MSC. (b) Variation of *n*_eff_ distribution of the LP_01_ mode in SMF and the OAM_1_ mode in RCF with the change of fiber core (ring core) radius. In experiment, when the pre-stretched diameter of SMF is 90.4 μm, MSC can generate the OAM_1_ mode. (c) Transmission spectra of the MSC in 965–985 nm, in which the transmission powers of the OAM_1_ and LP_01_ modes are close to −3 dB and below −19 dB.

For flexible generation of OAM modes, we employ a mode selective coupler (MSC) to realize mode conversion, as shown in Figure 6(a). The MSC operating at 980 nm is fabricated with the homemade ring-core fiber (RCF, detailed parameters in [40]) and SMF utilizing the fused biconical taper technology (detailed fabrication process in the Ref [41]). According to the coupled mode theory [42], LP_01_ mode in the SMF can be converted to OAM_1_ mode in the RCF when the two modes satisfy the phase-matching condition in the MSC, i.e., having the same *n*_eff_ [43]. It can be controlled by changing the stretched length. Figure 6(b) shows the variation of *n*_eff_ of LP_01_ mode in the SMF and OAM_1_ mode in the RCF, calculated as a function of the radius (*R*) of fiber core (or ring core). By calculation, the required pre-tapered diameter of the SMF is to be D_SMF_ = 87.5 μm (*ρ* = *R*_SMF_/*R*_RCF_ = 1.75/2.5 = 0.7, *D*_SMF_ = *ρ* × 125 μm = 87.5 μm). In the experiment, the pre-tapered SMF diameter approximately 90.4 μm is used to generate the first-order.

OAM mode. Figure 6(c) shows the transmission spectra of the MSC in the range of 965–985 nm. The maximum transmission power of the OAM_1_ mode in MSC is close to −3 dB, and the corresponding LP_01_ mode is attenuated below −19 dB. The high power extinction ratio indicates high mode conversion efficiency from LP_01_ to OAM_1_ mode, suggesting that the setup can be employed as a mode selective pump (MSP) used in the entire system.

### 4.2 Broad-spectrum fluorescence light source with OAM emission

**Figure 7:**
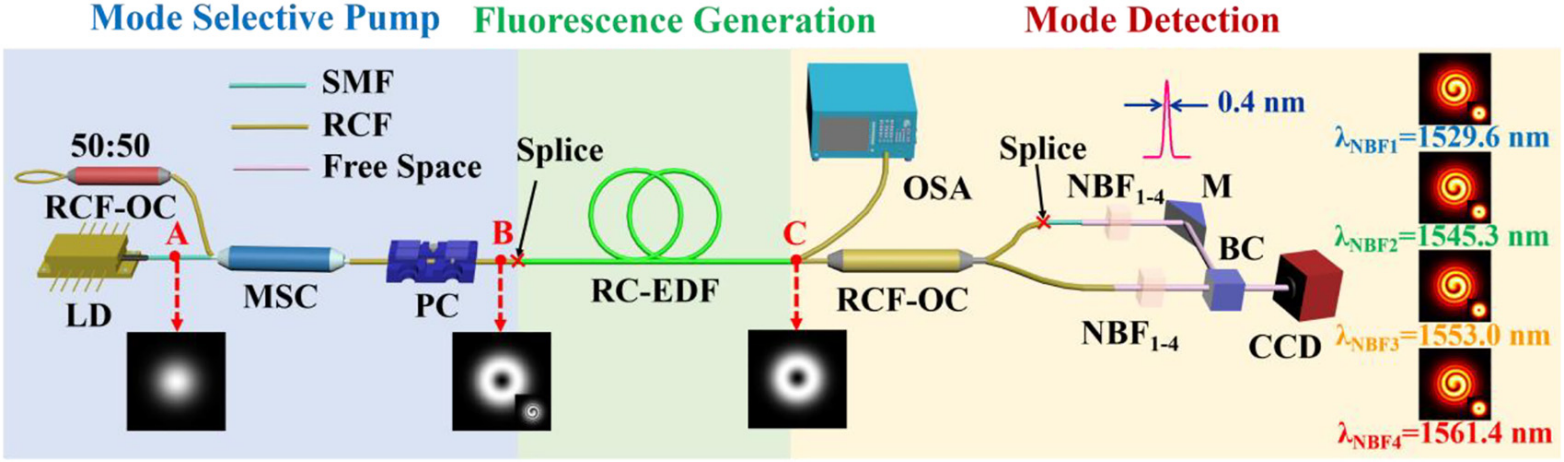
Schematic diagram of an all-fiber system of the OAM mode fluorescence emission, spectral analysis and mode detection. LD: laser diode; OC: optical coupler; MSC: mode selective coupler; PC: Polarization controller; RC-EDF: ring-core erbium-doped fiber; NBF: narrow-band filter; M: mirror; BC: beam combiner. CCD: charge-coupled device. NBFs with a band width of 0.4 nm are employed to select the narrow-band beam at the wavelengths of 1529.6, 1543.3, 1553.0, and 1561.4 nm.

Here, we present a straightforward method that generates the OAM-mode fluorescence with the in-house made RC-EDF. The schematic of our method represents a typical all-fiber configuration, as shown in Figure 7. The laser diode (LD) with output wavelength of ∼980 nm is used as a pump source. The MSC is used to convert LP_01_ mode to OAM mode, and the state of polarization of its output is controlled by a polarization controller (PC). A 50:50 RCF-optical coupler (OC) acts as a reflection mirror to reflect the backward transmission of light. An RC-EDF is used as a gain medium. Charge-coupled device (CCD, HAMAMATSU C10633, Japan) camera is used to observe the mode patterns in real time.

In the left part of experimental setup, the first-order OAM mode can be generated by the MSC based on the LP_01_ mode at position A (Figure 7). As an MSP, the excited OAM_1_ mode at position B can be selected and launched into a 1.6 m RC-EDF and further detected at position C. Therefore, the fluorescence emission can stably transmit in the form of ring-shaped modes.

**Figure 8:**
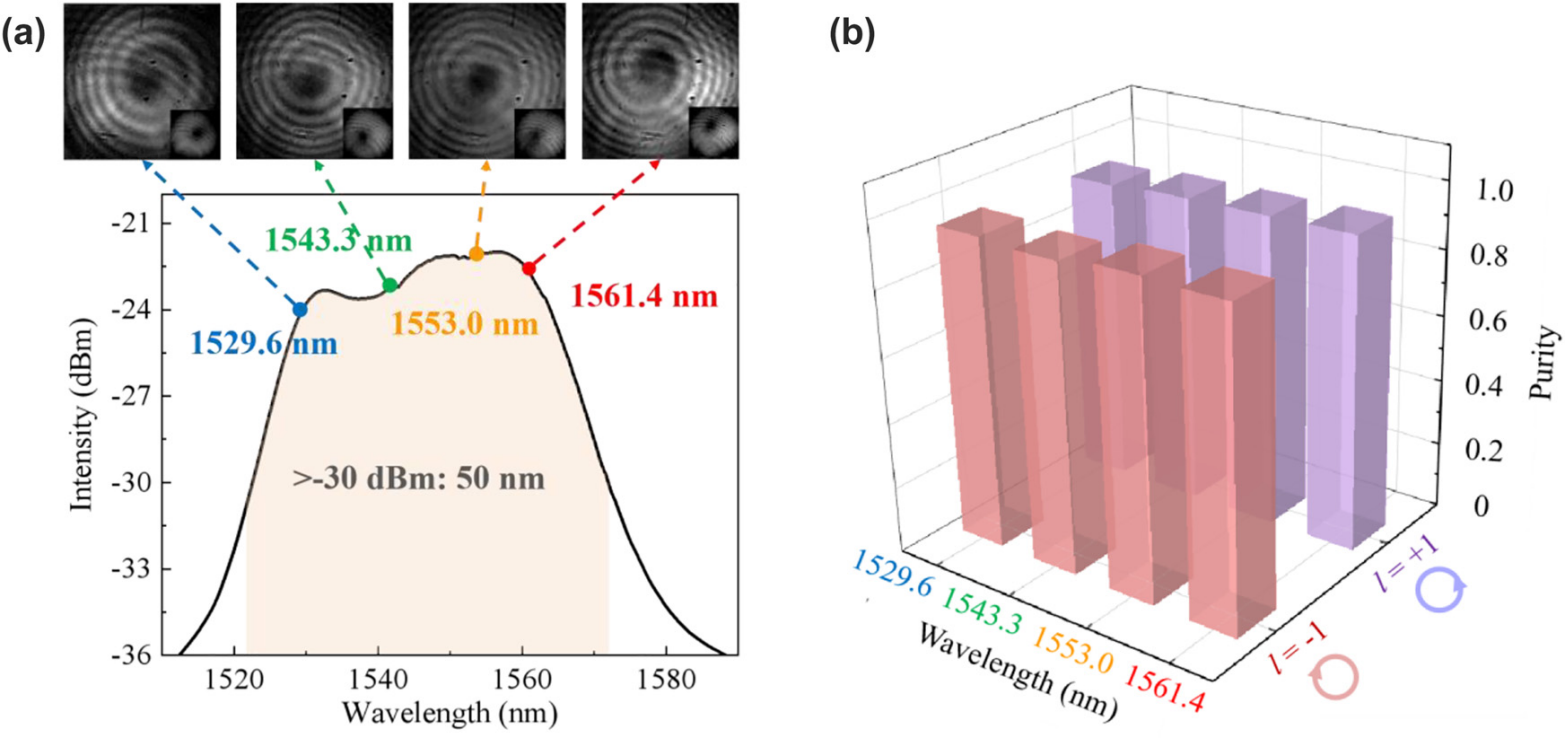
Fluorescence characteristics of the OAM-based light source. (a) OAM fluorescence spectrum. The tops are the mode patterns and spiral-shaped interferograms of the first-order OAM modes. (b) The first-order OAM modes purity at 1529.6, 1543.3, 1553.0, and 1561.4 nm with topological charge *l* = −1 and +1.

Furthermore, we build the spectral analysis and mode detection scheme, as shown in Figure 6. The fluorescence spectrum is recorded by an optical spectrum analyzer (OSA, YokogawaAQ-6370C, Japan), as shown in Figure 8(a). When the MSP provides the first-order OAM mode pump power of ∼180 mW, the maximum intensity of OAM mode fluorescence spectrum is approximately −22.0 dBm. The flat spectrum has a width of 50 nm from 1522 to 1572 nm with the intensity >−30 dBm. Therefore, this system allows the fluorescence to operate in a broadly spectral regime with high output power and flat spectrum, wherein the emission would be obtained within a specific OAM mode.

In order to illustrate that the high-order-mode fluorescence do indeed carry OAM, an interferometric detection setup is employed in the system. An RCF-OC is used to maintain the OAM mode transmission, from whose output beam is separated into two branches: one is to transmit high-order mode, and the other is directly spliced to a small section of SMF to convert the high-order mode to LP_01_ mode as a reference beam. Four pairs of narrow-band filters (NBFs) with a bandwidth of 0.4 nm are exploited to pick out the narrow-band beam at the wavelengths of 1529.6, 1543.3, 1553.0, and 1561.4 nm. The beam combiner (BC) and the reflecting mirror (M) are used to superimpose the beams of two paths with the same power and wavelength. The observed mode patterns and single-spiral interferograms of the first-order OAM mode at four different wavelengths are shown in the top of Figure 8(a). Meanwhile, the mode purity is used to scale the proportion of OAM mode and is measured by the method of spatial interferometry [44]. The first-order OAM mode purity obtained are 94.0%, 93.0%, 95.1%, and 94.7% across the four wavelengths from 1529.6 nm to 1561.4 nm, respectively. These results regarding the fluorescence emission of the *l* = −1 OAM mode suggest that our all-fiber system can intelligibly generate high-purity modes.

The study above is mainly based on the vortex beam with topological charge *l* = −1. Similarly, the case of *l* = +1 has also been explored. The topological charge of the generated OAM fluorescence can keep consistent with that of the pump beam, that is, under the pump beam at 980 nm with OAM of *l* = −1 or +1, the fluorescent characteristics, i.e., the observed mode patterns, interference patterns, and their detailed comparisons with the theoretical patterns at 1529.6, 1543.3, 1553.0, and 1561.4 nm, carry OAM of *l* = −1 or +1, respectively, as shown in Table 1. Specifically, the high purity of different *l* is realized at each detected wavelength, which is above 93%, as shown in Figure 8(b). Vortex displacement can be seen in these experimental images. The main reason is the structure and RI distribution of the RCF. In the RI profile of the RC-EDF (Figure 2(a)), the core-cladding RI difference is 0.0026, which means the fiber is not ring-shaped enough. Thus, it will greatly affect the OAM mode purity. The second reason is probably the mode-field unmatching of the RCF with the RC-EDF in the setup. Thirdly, the bandwidth of the NBFs employed is ∼0.4 nm, which is not narrow enough to present clear interferograms. Those will affect the generated fluorescence and the mode-pattern quality. The realization of this kind of OAM-based broad-spectrum light source can expound and verify the OAM-fluorescence-emission model mentioned in Figure 3, i.e., the donut-shaped distribution fluorescence emission can be achieved with a spiral phase wavefront in an RC-EDF. Meanwhile, the results can further demonstrate that the OAM-carrying photons will excite electrons and transfer the corresponding OAM to them in an active vortex field, and the released photons will also carry OAM with the same topological charge.

**Table 1:** Fluorescent characteristics at 1529.6, 1543.3, 1553.0, and 1561.4 nm generated by *l* = −1 and +1 pumps at 980 nm.

Pump with OAM	*λ* (NBF_1-4_) (nm)	Fluorescent characteristics	Purity of fluorescence	Observed modes & interferograms
*l* = −1	*λ* (NBF_1_)	*l* = −1	94.0%	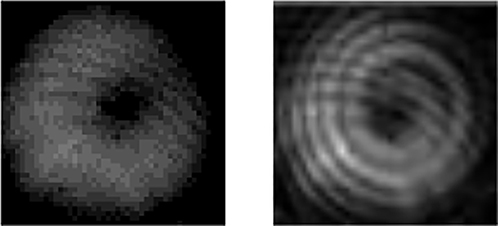
*l* = +1	1529.6	*l* = +1	92.4%	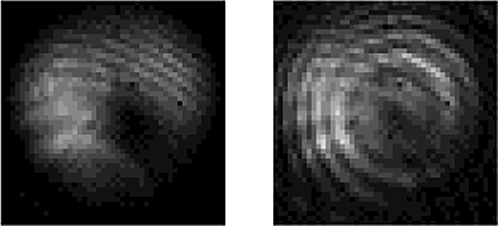
*l* = −1	*λ* (NBF_2_)	*l* = −1	93.0%	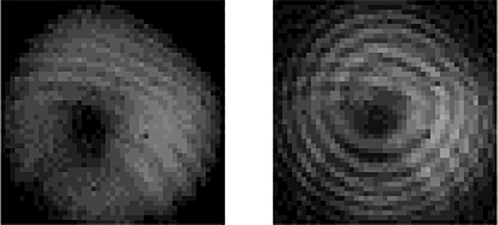
*l* = +1	1543.3	*l* = +1	93.7%	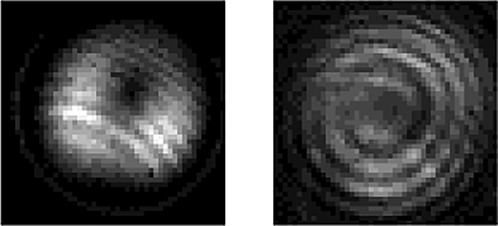
*l* = −1	*λ* (NBF_3_)	*l* = −1	95.1%	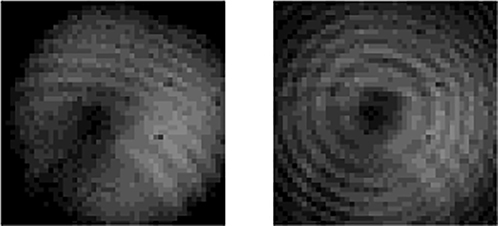
*l* = +1	1553.0	*l* = +1	94.0%	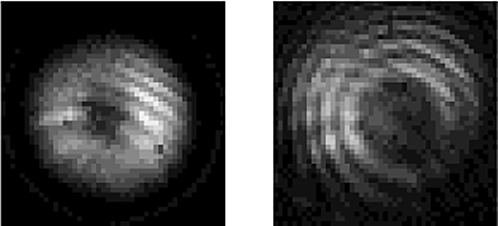
*l* = −1	*λ* (NBF_4_)	*l* = −1	94.7%	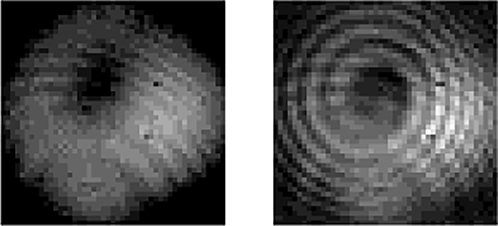
*l* = +1	1561.4	*l* = +1	94.3%	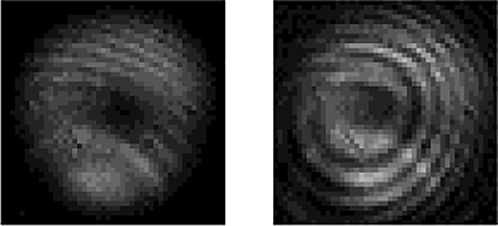

## 5 Conclusions

In conclusion, we construct a model of photon-electron interaction in an active optical fiber, and introduce a novel OAM-based fluorescence light source to demonstrate it experimentally. We first fabricate a 980 nm MSC to generate the first-order OAM mode using the passive RCF and the SMF, based on which the MSP is constructed to pump the active RC-EDF. The combined effect of the OAM-carrying photons in pump beam and electrons of erbium ions in a vortex field of the RC-EDF results in OAM fluorescence emission. We obtain the OAM mode fluorescence spectrum with the maximum intensity of −22.0 dBm. The width of fluorescence spectrum is up to 50 nm measured at the intensity greater than −30 dBm. Furthermore, four individual wavelengths, i.e., 1529.6, 1543.3, 1553.0, and 1561.4 nm, are extracted from the broad spectrum, which have been confirmed to possess the OAM characteristics, and whose purities of first-order OAM mode are above 93%. Based on the active fiber with a ring-core structure to directly generate OAM fluorescence emission, the methods described here could be used to develop an OAM-based light source. Moreover, we employ the established model to elucidate microscopically and macroscopically, which contains two processes: one is the excitation of OAM-carrying photons based on the interaction between photons and electrons; the other is the realization and transmission of the fluorescence with a ring-shaped distribution in a vortex field with a spiral phase wavefront in the active fiber. The OAM-based fluorescence light sources might pave the way to new approaches in high-capacity optical communication and high-sensitivity sensing, and enable quantum information technologies with encoded information beyond one bit per single photon, and high-resolution imaging with potentially increased spatial resolution beyond the diffraction limit, etc.
